# Supplementary material for: Experimental evolution partially restores functionality of bacterial chemotaxis network with reduced number of components
Source: PLoS Genet. 2025 Jul 10;21(7):e1011784. doi: 10.1371/journal.pgen.1011784 (PMC12270135; doi:10.1371/journal.pgen.1011784)
Supplement: S1 Text — (PDF) [file pgen.1011784.s001.pdf]

S1 Text

Computer simulations of the chemotactic response of evolved *cheR* strain

We simulate a population of running and tumbling agents in a two-dimensional rectangular channel ($L_x \times L_y$) with open ends in the x-direction and solid-wall boundary conditions on the y-axis (Fig 7A). A gradient of attractant (MeAsp) spans across the channel, so that the concentration of the attractant is:

$$c(x) = \begin{cases} c_0\left(\frac{1}{2} + g\left(x - \frac{L_x}{2}\right)\right), & \frac{L_x}{2} - \frac{1}{2g} \leq x \leq \frac{L_x}{2} + \frac{1}{2g} \\ 0, & x < \frac{L_x}{2} - \frac{1}{2g} \\ c_0, & x > \frac{L_x}{2} + \frac{1}{2g} \end{cases} \quad (1)$$

Each cell has a swimming speed v_i, drawn from a Gaussian distribution (mean $v_0 = 25$ µm/s, standard deviation $dv_0 = 2.5$ µm/s) reflecting experimental measurements, a swimming direction $\boldsymbol{u} = (\cos\theta, \sin\theta)$, a tumbling rate skewness s that depends on the current concentration c, and a binary run state r. The skewness s obeys:

$$s = \frac{1}{m}\left(1 + \frac{c}{K}\right)^H \quad (2)$$

and the cell tumbling bias is given by

$$b = \frac{1}{1+s} \quad (3)$$

In the case of the homogeneous population model, (m, K, H) are three constants fitted on the data presented in S10A Fig. In the heterogeneous population case, H is a constant, m is taken from a truncated Gaussian distribution (mean m_0, standard deviation σ_m, only positive m accepted) and K from a lognormal distribution ($K = K_0 \exp(\sigma_K \epsilon)$, with ϵ being a random number taken from a normal distribution of zero mean and unit variance). The parameter H was fixed arbitrarily, because we do not have a direct way of accessing it, and m and K distributions were adjusted in such a way that the simulated distributions of tumbling bias at various concentrations and the average bias dependence in concentration match relatively well with the experiments (Figs 7 and S10). Due to inefficiency of fit convergence, the parameters of the (m,K) distributions were chosen manually. These parameters are thus purely phenomenological. They cannot be linked to any underlying biological quantities, and in the case of the heterogeneous population, can only lead to a qualitative understanding of the effect of a large distribution of sensitivities.

When implemented, very slow adaptation in R1 was accounted for via a time dependent skewness $s(t)$. We found that the adaptation dynamics observed in Fig 6 was well modeled by using $s(t) = w(t)(1 + c(t)/K)^H$ with H, K and $w(0) = 1/m$ taken as in the non-adapting case, $c(t)$ the currently experienced attractant concentration and an adaptation of the skewness $s(t)$ towards $1/m$ via first order kinetics of $w(t)$:

$$\frac{dw}{dt} = r_a\left(\frac{1}{m}\frac{1}{\left(1+\frac{c(t)}{K}\right)^H} - w(t)\right) \tag{4}$$

where the adaptation rate r_a was fitted on the experimental data with $1/r_a \simeq 90$ min, which was independent of c and of whether a homogeneous or a heterogeneous population model was used.

Note that our calibration tracking data were obtained in homogeneous systems (no gradients of MeAsp) in two different suspending media, either our classical motility buffer (MB), which contains glucose, or glucose-free MB, to which 0, 50 µM or 500 µM MeAsp were added (S10 Fig). For each condition, the tracking datasets combine two sub-datasets taken close to and away from the surface. We used the datasets of both conditions to calibrate the model, allowing only the m-parameters to vary with glucose concentration, while H and the K-parameters are independent of it. Since the same constant background glucose concentration is present in the experiments with gradients, we used only the corresponding parameters in the agent-based simulations.

The cell state r transitions from "run" ($r = 1$) to "tumble" ($r = 0$) at a rate $s(c)/\tau_0$ and from "tumble" to "run" at $1/\tau_0$, with τ_0 chosen to match the mean tumble duration.

Cells are always propelled at v_i, but experience a run state dependent reorientation rate, modeled by a rotational diffusion coefficient:

$$\frac{d(x_i, y_i)}{dt} = v_i(\cos\theta_i, \sin\theta_i) \tag{5}$$

$$d\theta_i = \sqrt{2Ddt}\eta \tag{6}$$

With $D = D_r + (1-r)D_T$ accounting for rotational Brownian motion (D_r) and reorientation during tumbles (D_T), η a Gaussian random variable with unit variance and zero mean, and dt the simulation step.

Collision with walls in the y-direction are handled via a quadratic-potential repulsion generating both a force and a torque on the bacterium, so that for a penetration into the wall Δy:

$$\left(\frac{dy_i}{dt}\right)_{wall} = -v_i \sin\theta_i - k_s\Delta y \tag{7}$$

$$\left(\frac{d\theta_i}{dt}\right)_{wall} = -k_s|\Delta y| \cos\theta_i \sin\theta_i \tag{8}$$

The x-boundaries of the simulation box are absorbing sinks and constant sources for the cells. A cell trajectory ends if its position $x_i(t) > L_x$ or $x_i(t) < 0$. In addition to the cells that spawn at the start of the simulation in the simulation box with uniform distribution of position and orientation, new cells are spawned at both ends of the channel with a rate $1/\tau_{spawn} = 2\rho L_y v_0/\pi$, where ρ is the cell density, which therefore stays constant throughout the simulation in absence of gradients. Cells spawned with a downward orientation ($u_x < 0$) appear at the top-end of the channel (close to $x = L_x$), and at the bottom end for an upward orientation. The cell motion is solved by Euler integration with a time step dt during a total simulation time T, with all cell parameters being saved every n^{th} simulation step for memory management reasons. Used simulation parameters are listed in Table A.

To mimic our experimental measurements, we measure the population-averaged drift and all other cell behavior characteristics only in a 700-µm-wide box around the center of the channel, after 200 s of initial simulation beyond which these quantities reach steady state. We performed 9 independent simulations, each producing about 1900 detected trajectories, for around 4×10^7 ndt of total trajectory duration per condition.

Table A. Parameters of homogeneous and heterogeneous models used in simulations.

Name/symbol	Homogeneous model	Heterogeneous model
L_x	2048 µm	
L_y	512 µm	
g	0.0005 µm^{-1}	
dt	0.01 s	
T	500 s	
n	10	
ρ	0.01 cells.µm^{-2}	
c_0	[0; 50; 500] µM	
**Cell parameters**		
v_0	25 µm/s	
dv_0	2.5 µm/s	
D_r	0.1 s^{-1}	
D_T	12 s^{-1}	
k_s	50 s^{-1}	
$1/\tau_0$	6 s^{-1}	
r_a	0 (non-adaptive) or 1/90 min^{-1} (adaptive)	
H	0.8	10
m_0	0.37	0.2
σ_m	0	0.5
K_0	33 µM	750 µM
σ_K	0	1.5
m_0 (MB - Glucose)	0.70	0.75
σ_m (MB - Glucose)	0	0.45
