# Supplementary figures and images for: Experimental evolution partially restores functionality of bacterial chemotaxis network with reduced number of components
Source: PLoS Genet. 2025 Jul 10;21(7):e1011784. doi: 10.1371/journal.pgen.1011784 (PMC12270135; doi:10.1371/journal.pgen.1011784)

A

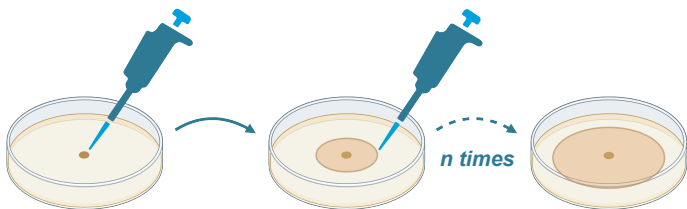

B

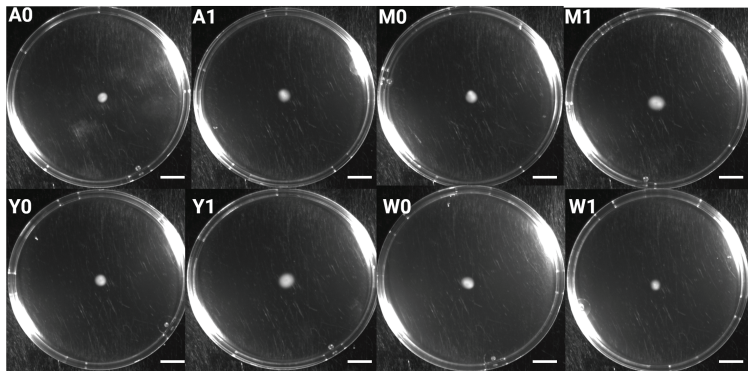

Supplement: S1 Fig — (A) A schematic of the evolution experiment. The cells demonstrating the fastest spreading were collected from the edge of the spreading ring and inoculated in the middle of a fresh TBSA plate. This procedure was repeated for 30 days. Created with BioRender. (B) Spreading of ∆cheA (A), ∆cheY (Y), ∆mcp (M) or ∆cheW (W) deletion strains, either before (denoted as “0”) or after evolution for 30 days in soft agar. An example of one evolved line (denoted as “1”) is shown for each strain. Scale bars are 2 cm. (PDF) [file pgen.1011784.s002.pdf]

A

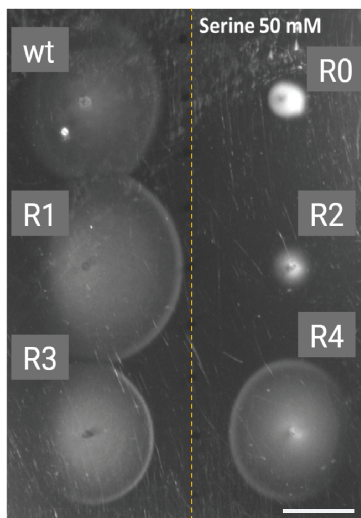

B

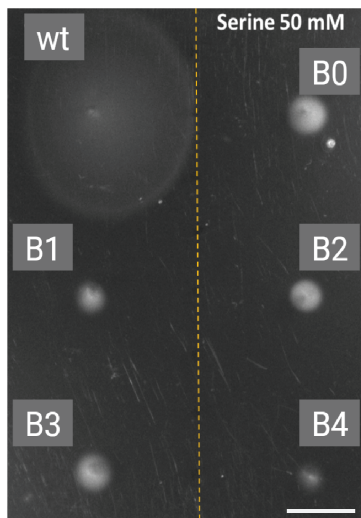

C

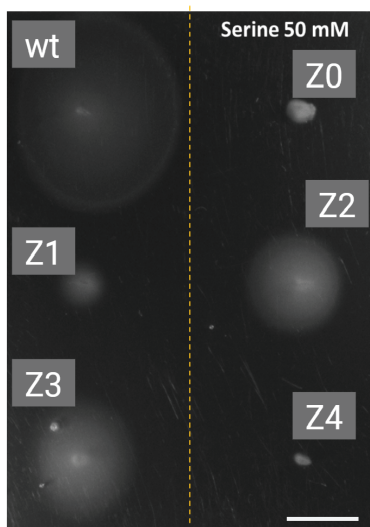

D

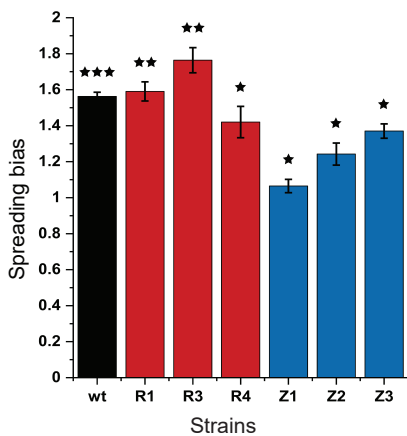

Supplement: S6 Fig — (A-C) Indicated strains were tested for biased spreading on M9 minimal medium soft-agar (M9SA) plates with a pre-established gradient of serine (50 mM at the source). Scale bars represent 1 cm. (D) Spreading bias was quantified as the ratio between the distances from the inoculation point of the expanding colony to its edges up and down the gradient. The measurements were performed in three independent replicates; error bars indicate standard errors of the mean. Significance analysis was done in comparison to 1 (no bias). P values were calculated using one-tailed Student t-test (ns, not significant; *, P < 0.05; **, P < 0.01; ***, P < 0.001). (PDF) [file pgen.1011784.s007.pdf]
