# Supplementary material for: Experimental evolution partially restores functionality of bacterial chemotaxis network with reduced number of components
Source: PLoS Genet. 2025 Jul 10;21(7):e1011784. doi: 10.1371/journal.pgen.1011784 (PMC12270135; doi:10.1371/journal.pgen.1011784)
Supplement: S1 Table — (PDF) [file pgen.1011784.s012.pdf]

S1 Table. Mutations identified in evolved Δ*cheR* lines.

Gene	Annotation[a]	R1	R2	R3	R4	R5	R6	R7	R8
tsr	N54D			■					
tsr	V199M								■
tsr	M249T		■						
tsr	L263M				■				
tsr	T305M	■							
tsr	G426E						■		
tsr	T441K					■			
tar	A166P			■					
tar	V433I							■	
tar	Q502 frameshift								■
cheZ	Q64L						■		
cheZ	Q204L	■							
cheB	S8P						■		
cheB	L71F				■				
cheB	R75C	■							
cheB	A130V								■
cheB	R257G		■						
cheB	G313C					■			
motA	L2F				■				
fliF	G520R		■						
fliG	E114Q			■					
fliG	G165S								■
fliG	G165A					■			
fliI	T42I								■
fliI	M80I				■				
fliI	M178I	■							
fliI	R197H							■	
fliM	V98L							■	
fliM	P195L			■					
rsfS / *cobC*	IS1				■				
lysO	P241L			■					
ycjM	IS1		■						
ddpB	I6T	■							
rsmI	P101L		■						
nanA–sspA	Δ4,084 bp			■					
sspA	14 bp insertion	■							
atpI / *rsmG*	IS1		■						
fabR	IS1				■				
perR	IS1								
gltI	IS5					■			
mngB / *cydA*	IS5					■			
opgG	T405K							■	
purR	Δ20 bp								
eda	W4C						■		
edd	5 bp insertion							■	
edd	S16*					■			
rbn	IS1							■	
ppk	IS1							■	
parC	A414S						■		
sspA	IS1					■			
gntT	IS1								■
rplI	Q133*						■		

[a]Amino acid substitution is indicated where relevant.
*: Nonsense mutation (stop codon).
IS1 or IS5: Mutation introduced by insertion sequence.
See S1 Data for the exact list of mutations.
