# Supplementary material for: Experimental evolution partially restores functionality of bacterial chemotaxis network with reduced number of components
Source: PLoS Genet. 2025 Jul 10;21(7):e1011784. doi: 10.1371/journal.pgen.1011784 (PMC12270135; doi:10.1371/journal.pgen.1011784)
Supplement: S2 Table — (PDF) [file pgen.1011784.s013.pdf]

S2 Table. Mutations identified in evolved Δ*cheB* lines.

Gene	Annotation[a]	B1	B2	B3	B4
tsr	A94T				
tsr	S437R				
tap	S428L				
tar	A411T				
tar	A496-A498 deletion				
cheW	D139(ALGD insertion)				
cheA	E319A				
fliN	A115S				
rpsA	R86H				
yciW	E217K				
ydcO	IS1				
ddpB	I6T				
fadD	IS5				
fadD	76 bp deletion				
atpD	L163R				
atpA	D289H				
atpH	G84H				
atpI / *rsmG*	IS5				
atpI / *rsmG*	IS1				

[a]Amino acid substitution is indicated where relevant.

IS1 or IS5: Mutation introduced by insertion sequence.

See S1 Data for the exact list of mutations.
