# Supplementary material for: Experimental evolution partially restores functionality of bacterial chemotaxis network with reduced number of components
Source: PLoS Genet. 2025 Jul 10;21(7):e1011784. doi: 10.1371/journal.pgen.1011784 (PMC12270135; doi:10.1371/journal.pgen.1011784)
Supplement: S3 Table — (PDF) [file pgen.1011784.s014.pdf]

S3 Table. Mutations identified in evolved Δ*cheZ* lines.

Gene	Annotation[a]	Z1	Z2	Z3	Z4
tar	S31 frameshift				
tar	Q155*				
cheA	L92W				
cheA	M98V				
cheA	P457L				
cheA	D476E				
clpX	IS1				
rhsC	A598 frameshift				
opgH	V463G				
topA	L781Q				
bamD	Y205H				
rpoD	27 bp insertion				
atpD	L163R				
atpI / *rsmG*	IS5				

[a]Amino acid substitution is indicated where relevant.

*: Nonsense mutation (stop codon).

IS1 or IS5: Mutation introduced by insertion sequence.

See S1 Data for the exact list of mutations.
