# Supplementary material for: Experimental evolution partially restores functionality of bacterial chemotaxis network with reduced number of components
Source: PLoS Genet. 2025 Jul 10;21(7):e1011784. doi: 10.1371/journal.pgen.1011784 (PMC12270135; doi:10.1371/journal.pgen.1011784)
Supplement: S4 Table — (PDF) [file pgen.1011784.s015.pdf]

S4 Table. Strains and plasmids used in this study.

**Name**		**Source**
**Strains**	**Strain genetic background**	
RP437 (WT)	*Escherichia coli* RP437 (wild type for chemotaxis)	(1)
VS126(R0)	RP437 Δ*cheR*	(2)
RP4972(B0)	RP437 Δ*cheB*	(1)
VS161(Z0)	RP437 Δ*cheZ*	(2)
VS166(A0)	RP437 Δ*cheA*	(3)
VS100(Y0)	RP437 Δ*cheY*	(4)
VS289(W0)	RP437 Δ*cheW*	(1)
UU1250(M0)	RP437 Δ*tar* Δ*tap* Δ*tsr* Δ*aer* Δ*trg*	(5)
VS1934(WT *tsr**)	RP437 *tsr** (T305M)	This study
VS1935(R0 *tsr**)	RP437 Δ*cheR tsr** (T305M)	This study
VS1936	R1 Δ*cheB*	This study
VS1956(R0 *cheB**)	RP437 Δ*cheR cheB** (R75C)	This study
VS1957(WT *cheB**)	RP437 *cheB** (R75C)	This study
VS1958(WT *cheZ**)	RP437 *cheZ** (Q204L)	This study
VS1959(R0 *cheZ**)	RP437 Δ*cheR cheZ** (Q204L)	This study
VS1960(R0 *tsr*cheZ**)	RP437 Δ*cheR tsr** (T305M) *cheZ** (Q204L)	This study
VS1961(R0 *cheB*cheZ**)	RP437 ΔcheR *cheB** (R75C) *cheZ** (Q204L)	This study
VS1962(R0 *cheB*tsr**-R4)	RP437 Δ*cheR cheB**(L71F) *tsr** (L263M)	This study
VS1963(R0 *cheB*tsr**-R5)	RP437 Δ*cheR cheB** (G313C) *tsr** (T441K)	This study
VS1964(WT *fliI**)	RP437 *fliI** (M178I)	This study
VS1965(R0 *fliI**)	RP437 Δ*cheR fliI** (M178I)	This study
VS1966(R0 *tsr*cheZ*fliI**)	RP437 Δ*cheR tsr**(T305M) *cheZ** (Q204L) *fliI** (M178I)	This study
VS1968(R0 *tsr**-R4)	RP437 Δ*cheR tsr** (L263M)	This study
VS1970(R0 *tsr**-R5)	RP437 Δ*cheR tsr** (T441K)	This study
VS1972(R0 *cheB**-R4)	RP437 Δ*cheR cheB** (L71F)	This study
VS1974(R0 *cheB**-R5)	RP437 Δ*cheR cheB** (G313C)	This study
VS1975(R0 *tsr*cheB**)	RP437 Δ*cheR tsr** (T305M) *cheB**(R75C)	This study
VS1976(R0 *tsr*cheB*cheZ**)	RP437 Δ*cheR tsr** (T305M) *cheZ** (Q204L) *cheB** (R75C)	This study
VS1977(R0 *tsr*cheB*cheZ*fliI**)	RP437 Δ*cheR tsr** (T305M) *cheZ** (Q204L) *fliI** (M178I) *cheB** (R75C)	This study
VS2161(R0 *tsr*cheB*cheZ*fliI*sspA**)	RP437 Δ*cheR tsr** (T305M) *cheZ** (Q204L) *fliI** (M178I) *cheB** (R75C) *sspA** (V10 frameshift)	This study
**Plasmids**	**Details**	
pVS88	CheY-YFP / CheZ-CFP expression plasmid used for FRET assays	(6)
pAM109	GFP reporter for *fliC* promoter was constructed based on pUA66	(7)
pKD46	Used for making chromosomal deletions of genes with FRT sites	(8)
pKD45	Used for SNP introduction; counterselection with *ccdB* gene under a rhamnose-inducible promoter while introducing	J.S.Parkinson, personal gift and (8)

Supplementary References

1. J. S. Parkinson, Complementation analysis and deletion mapping of *Escherichia coli* mutants defective in chemotaxis. *J Bacteriol* **135**, 45-53 (1978).
2. L. Løvdok, M. Kollmann, V. Sourjik, Co-expression of signaling proteins improves robustness of the bacterial chemotaxis pathway. *J Biotechnol* **129**, 173-180 (2007).
3. D. Kentner, V. Sourjik, Dynamic map of protein interactions in the *Escherichia coli* chemotaxis pathway. *Mol Syst Biol* **5**, 238 (2009).
4. V. Sourjik, H. C. Berg, Localization of components of the chemotaxis machinery of *Escherichia coli* using fluorescent protein fusions. *Mol Microbiol* **37**, 740-751 (2000).
5. P. Ames, C. A. Studdert, R. H. Reiser, J. S. Parkinson, Collaborative signaling by mixed chemoreceptor teams in *Escherichia coli. Proc Natl Acad Sci U S A* **99**, 7060-7065 (2002).
6. V. Sourjik, H. C. Berg, Functional interactions between receptors in bacterial chemotaxis. *Nature* **428**, 437-441 (2004).
7. B. Ni *et al.*, Evolutionary Remodeling of Bacterial Motility Checkpoint Control. *Cell Rep* **18**, 866-877 (2017).
8. K. A. Datsenko, B. L. Wanner, One-step inactivation of chromosomal genes in *Escherichia coli* K-12 using PCR products. *Proc Natl Acad Sci U S A* **97**, 6640-6645 (2000).
