# Supplementary material for: Experimental evolution partially restores functionality of bacterial chemotaxis network with reduced number of components
Source: PLoS Genet. 2025 Jul 10;21(7):e1011784. doi: 10.1371/journal.pgen.1011784 (PMC12270135; doi:10.1371/journal.pgen.1011784)
Supplement: Attachment — Submitted filename: Sourjik review 2025.docx [file pgen.1011784.s018.docx]

The authors carried out a very thorough investigation of the ability to “evolve” restored chemotaxis function for strains deleted for one of the six Che genes (CheA, CheB, CheR, CheW, CheY, and CheZ) or all four (or five if Aer was included) chemoreceptors of *E. coli*. As far as I can tell, no artificial mutagenesis was conducted.

All four replicates of 30 rounds of selection for increased spreading in tryptone soft agar of the Δ*cheR*, Δ*cheB*, and Δ*cheZ* evolved lines that showed increased spreading. In contrast, none of the four replicates with Δ*cheA*, Δ*cheW*, Δ*cheY*, Δ*cheRB*, or Δreceptor strains evolved lines with increased spreading. The result with the Δ*che*A, Δ*cheW*, Δ*cheY*, and Δreceptor is expected, as they do not make CheY-P, which is essential for CW flagellar rotation of the wild-type motor. Thus, they are locked in CCW rotation, or smooth swimming, and cannot spread in soft agar because they get trapped in cul de sacs in the agar mesh. The result with the Δ*cheRB* strain is a bit more difficult to understand on face value, because they should be able to tumble.

Δ*cheR* strains do not spread because they also turn their flagella almost exclusively CCW and swim smoothly. Δ*cheB* and Δ*cheZ* strains do not spread because they turn their flagella predominately CW and thus never form a coherent bundle of left-handed helical flagellar filaments that can make the cells swim in a translational fashion (run). However, they have the gene products required to make CheY-P, and the control of the production of CheY-P by the chemotaxis system can be biased to make levels of CheY-P that allow the run-tumble behavior that is required for spreading in soft agar. Indeed, in all twelve of the evolved lines that showed spreading, four for each of Δ*cheR*, Δ*cheB*, and Δ*cheZ*, the cells were seen to run and tumble.

The most interesting result was with the lines derived from ΔcheR. At least the R1 line not only spread, it also showed a sharp ring at the edge of the spreading colony, which is characteristic of the ability to carry out the chemotaxis to L-serine mediated by the Tsr chemoreceptor. This indicates that cells in this line not only can run and tumble to spread in soft agar, they can also carry out chemotaxis. This was confirmed by showing that these cells can migrate up static concentration gradients generated in a flow device.

I have these observations.

1) The knowledge that cells that are missing essential components of the Che system can spread in soft agar if they can run and tumble is at least 40 years old, as the authors acknowledge. What is new here is that the authors have identified the mutations, typically multiple ones with additive effects, that restore the ability to run and tumble. They are precisely the mutations that one might have predicted. For the Δ*cheR* lines they are mutations that would be expected to increase CheY-P production by increasing CheA activity or increasing the ability of chemoreceptors to stimulate CheA activity. For the Δ*cheB* and Δ*cheZ* lines, they are mutations that should decrease the activity of CheA or the stimulation of CheA activity and thus the overproduction of CheY-P. In each case, running and tumbling is restored. The evolved lines also often contained mutations that increase flagellar gene expression, so that the cells swim faster.
2) The most interesting case is the derived lines from the ΔcheR mutant that can actually carry out chemotaxis to move in gradients of chemoattractants. It has been thought that the combined activities of CheR and CheB are required for cells to make the temporal comparisons in attractant concentration that allow them to move in chemical gradients. It would seem that CheR is not absolutely necessary for this. The authors provide a speculative mechanism and develop a computer program to test their model. Their program can get cells to move in chemical gradients, but not as well as the Δ*cheR* evolved lines can. To be flippant, they have shown that evolution can outperform computer programmers. I would like to see them develop a bit more completely and clearly their model for how the Δ*cheR* evolved line manages to carry out real chemotaxis. I suspect this involves the fact that receptors are synthesized with several of their methylatable glutamate residues as glutamines that are deamidated by CheB, which could provide a sort of one-shot adaptation for a receptor. The Δ*cheR* evolved strains all retain some CheB activity, and the Δ*cheRB* strain never evolved lines that can swarm. The residual activity of CheB in a Δ*cheR* strain can presumably still be regulated by phosphorylation of CheB by CheA, which suggests that enough adaptation mediated by changes in CheB activity may be possible to accomplish some degree of chemotaxis when cells are able to run and tumble.
3) My biggest criticism is that the authors seem to take credit for replicating millions of years of evolution that fine-tuned a chemotaxis system by evolving a different mechanism for chemotaxis through directed laboratory evolution over a month or two. I find that unjustified. What they have actually done is to remove a piece of a highly evolved and very adaptable chemotaxis signaling network and then restored a lesser level of function by multiple mutations in chemotaxis and flagellar genes that compensate for the loss of the removed component. The evolved systems still do not function as well as the wild-type system even under controlled laboratory conditions. I predict they would perform even less well than the wild-type system in the real world, where chemical gradients are transient in time and irregular in space. I think the authors should openly explain this and not claim to have generated sped-up evolution in a petri plate.
4) An analogy may reinforce my point from the previous paragraph. A person losing a leg will initially be virtually immobile. However, even without an artificial limb, that person will learn how to get around reasonably well by crawling, hopping, using a crutch, or finding some other means of non-bipedal locomotion. (Yes, now they could get a very sophisticated artificial leg.) I think it is inappropriate to say that they have evolved or developed a new means of locomotion, as all of those latent skills were present before the leg was lost. They just had not been required to be developed, but they could be refined and to some extent repurposed.
5) My conclusion from the points raised in the two preceding paragraphs is that the title and emphasis of the paper should changed, and its claimed impact be put into perspective. It is an inelegant alternative title, and something better wording can no doubt but found, but more accurate would be “Loss of Adaptive Methylation by CheR Can Be Partially Compensated by Multiple Mutations Targeting Other Components of the Chemotaxis Signaling Pathway.”
6) It is also misleading to say that the system has lower complexity. The compensation for the loss of CheR, CheZ, or CheB, came through the accumulation of multiple mutations in other components of the chemotaxis and flagellar system that had to be finely balanced and likely made the system far less robust and flexible. The newly evolved chemotaxis system is almost certainly inferior to the one it replaced. Simply removing one component does not make the system less complex if multiple changes in the remaining components are required to have it function even somewhat effectively. *Helicobacter pylori* can get by with a simpler system because it lives in a rather simple environment.

Minor editorial comments.

Line 565. The “circumvents” should be “circumvent.”

Lines 879-880. Rewrite “…where the corresponding individual chemotaxis gene was deleted, either before…or after evolution for 30 days.” The gene was not deleted before or after 30 days of evolution. The evolved strain with the deleted gene was evaluated before and after evolution for 30 days.
