# Supplementary material for: Experimental evolution partially restores functionality of bacterial chemotaxis network with reduced number of components
Source: PLoS Genet. 2025 Jul 10;21(7):e1011784. doi: 10.1371/journal.pgen.1011784 (PMC12270135; doi:10.1371/journal.pgen.1011784)
Supplement: Attachment — Submitted filename: Response to Reviewers Kargeti et al.docx [file pgen.1011784.s020.docx]

**Response to Reviewers**

**Reviewer #1: *The authors carried out a very thorough investigation of the ability to “evolve” restored chemotaxis function for strains deleted for one of the six Che genes (CheA, CheB, CheR, CheW, CheY, and CheZ) or all four (or five if Aer was included) chemoreceptors of E. coli. As far as I can tell, no artificial mutagenesis was conducted.***

We thank the Reviewer for the detailed summary of our findings and for acknowledging that our investigation was thorough. It is correct that no artificial mutagenesis was conducted. We now mention this in the Results and Methods sections.

***All four replicates of 30 rounds of selection for increased spreading in tryptone soft agar of the ΔcheR, ΔcheB, and ΔcheZ evolved lines that showed increased spreading. In contrast, none of the four replicates with ΔcheA, ΔcheW, ΔcheY, ΔcheRB, or Δreceptor strains evolved lines with increased spreading. The result with the ΔcheA, ΔcheW, ΔcheY, and Δreceptor is expected, as they do not make CheY-P, which is essential for CW flagellar rotation of the wild-type motor. Thus, they are locked in CCW rotation, or smooth swimming, and cannot spread in soft agar because they get trapped in cul de sacs in the agar mesh. The result with the ΔcheRB strain is a bit more difficult to understand on face value, because they should be able to tumble.
ΔcheR strains do not spread because they also turn their flagella almost exclusively CCW and swim smoothly. ΔcheB and ΔcheZ strains do not spread because they turn their flagella predominately CW and thus never form a coherent bundle of left-handed helical flagellar filaments that can make the cells swim in a translational fashion (run). However, they have the gene products required to make CheY-P, and the control of the production of CheY-P by the chemotaxis system can be biased to make levels of CheY-P that allow the run-tumble behavior that is required for spreading in soft agar. Indeed, in all twelve of the evolved lines that showed spreading, four for each of ΔcheR, ΔcheB, and ΔcheZ, the cells were seen to run and tumble.***

We agree with this summary of our initial findings, and it is consistent with our presentation of the data in the manuscript. However, our data suggest that the ability to generate an intermediate level of CheY-P (and thus tumbles) is necessary but not sufficient condition to determine which mutants can recover. As mentioned by the Reviewer, one evidence for that is our observation that Δ*cheRB* strain did not recover spreading. Moreover, it is further exemplified by the evolution of Δ*cheB* strain, where the initial tumbling bias of the parental deletion strain was closer to the wildtype optimum than that of *ΔcheR* or *ΔcheZ*. In several cases, its spreading could improve even without changes in the tumbling bias. We now emphasize these points more explicitly in the Results and Discussion sections.

***The most interesting result was with the lines derived from ΔcheR. At least the R1 line not only spread, it also showed a sharp ring at the edge of the spreading colony, which is characteristic of the ability to carry out the chemotaxis to L-serine mediated by the Tsr chemoreceptor. This indicates that cells in this line not only can run and tumble to spread in soft agar, they can also carry out chemotaxis. This was confirmed by showing that these cells can migrate up static concentration gradients generated in a flow device.***

We fully agree with this assessment.

***I have these observations.
1) The knowledge that cells that are missing essential components of the Che system can spread in soft agar if they can run and tumble is at least 40 years old, as the authors acknowledge. What is new here is that the authors have identified the mutations, typically multiple ones with additive effects, that restore the ability to run and tumble. They are precisely the mutations that one might have predicted. For the ΔcheR lines they are mutations that would be expected to increase CheY-P production by increasing CheA activity or increasing the ability of chemoreceptors to stimulate CheA activity. For the ΔcheB and ΔcheZ lines, they are mutations that should decrease the activity of CheA or the stimulation of CheA activity and thus the overproduction of CheY-P. In each case, running and tumbling is restored. The evolved lines also often contained mutations that increase flagellar gene expression, so that the cells swim faster.***

We generally agree that the observed evolutionary adjustment of the tumbling bias to an intermediate level by selected mutations makes sense, and this is how the results are discussed in the manuscript. However, as we emphasize in the manuscript, the obtained mutations are more specific than that: although both *ΔcheB and ΔcheZ* mutants are tumbly, they acquire different sets of mutations that compensate this defect, and different mutants with a similar tumbling bias and swimming velocity spread differently. Moreover, not only R1 but most of the evolved R and Z lines exhibit biased spreading in gradients.

***2) The most interesting case is the derived lines from the ΔcheR mutant that can actually carry out chemotaxis to move in gradients of chemoattractants. It has been thought that the combined activities of CheR and CheB are required for cells to make the temporal comparisons in attractant concentration that allow them to move in chemical gradients. It would seem that CheR is not absolutely necessary for this. The authors provide a speculative mechanism and develop a computer program to test their model. Their program can get cells to move in chemical gradients, but not as well as the ΔcheR evolved lines can. To be flippant, they have shown that evolution can outperform computer programmers. I would like to see them develop a bit more completely and clearly their model for how the ΔcheR evolved line manages to carry out real chemotaxis. I suspect this involves the fact that receptors are synthesized with several of their methylatable glutamate residues as glutamines that are deamidated by CheB, which could provide a sort of one-shot adaptation for a receptor. The ΔcheR evolved strains all retain some CheB activity, and the ΔcheRB strain never evolved lines that can swarm. The residual activity of CheB in a ΔcheR strain can presumably still be regulated by phosphorylation of CheB by CheA, which suggests that enough adaptation mediated by changes in CheB activity may be possible to accomplish some degree of chemotaxis when cells are able to run and tumble.***

We agree that the role of such “one-shot” adaptation is a plausible hypothesis. It was already mentioned in the Discussion of the previous version of our manuscript that such “one-sided” irreversible adaptation, coupled to receptor expression, is likely important for adjusting the basal level of the pathway activity in the evolved strain dependent on the background stimulation, to enable bacteria to have the tumbling bias in the right range at a given background stimulation. Such adjustment was already implicitly included in our model, since we used the experimentally determined tumbling bias.

However, beyond such stimulation-dependent bias adjustment, it is less clear how such “one-sided” adaptation by gradual deamidation could enable chemotaxis. While it may contribute to bacterial spreading in soft agar, where new half-modified receptors are constantly produced at a high rate, it is less obvious whether it would enhance the chemotactic drift in the microfluidics experiments performed with non-growing cells where new protein production is very limited.

Moreover, in the conventional model of chemotaxis, the methylation-dependent temporal memory needs to be on the time scale of a bacterial run, requiring the adaptation to be quite fast, on the timescale of tens of seconds. Such fast adaptation in non-growing cells could not be observed in our FRET experiments. We now partly moved these data to the main text (new Figure 6B) and discussed them in greater detail.

We have also performed additional experiments to investigate the ability of R1 cells to adapt to a given background stimulation under conditions of our microfluidics experiments. We indeed observed a slow recovery of their tumbling bias, on the timescale of several hours. We now show these new data (new Figure 6E). Since this slow adaptation cannot function as a conventional short-term memory, it does not enhance the chemotactic drift in our simulations (new Figure 7D), but we hypothesize that it plays an important role in adjusting cell tumbling bias to an intermediate level at different background concentration of attractants. We elaborate on these points in a greater detail in the Results and Discussion of the revised manuscript.

***3) My biggest criticism is that the authors seem to take credit for replicating millions of years of evolution that fine-tuned a chemotaxis system by evolving a different mechanism for chemotaxis through directed laboratory evolution over a month or two. I find that unjustified. What they have actually done is to remove a piece of a highly evolved and very adaptable chemotaxis signaling network and then restored a lesser level of function by multiple mutations in chemotaxis and flagellar genes that compensate for the loss of the removed component. The evolved systems still do not function as well as the wild-type system even under controlled laboratory conditions. I predict they would perform even less well than the wild-type system in the real world, where chemical gradients are transient in time and irregular in space. I think the authors should openly explain this and not claim to have generated sped-up evolution in a petri plate.***

We never claimed that we could do better than millions of years of evolution, and we fully agree that there is only a partial restoration of chemotaxis in the absence of one of the components. It is also clear that the natural complete pathway must be better – otherwise its components would have been naturally lost. One would indeed not expect that the evolved strains will be comparably efficient as the wildtype under all conditions, since they only evolved under specific selection. But although selected for spreading on soft-agar plates, the evolved R1 strain also exhibits a rather efficient taxis in microfluidic gradients in liquid, suggesting that the selection for chemotaxis was more general.

Having said that, it is nevertheless striking and highly surprising that ANY restoration of chemotaxis upon a loss of the highly conserved component(s) could be achieved in such short evolutionary term. We now expanded the Discussion to better address these points. We have also clearly stated now in the Abstract that the evolved strategy is less efficient than the original one.

***4) An analogy may reinforce my point from the previous paragraph. A person losing a leg will initially be virtually immobile. However, even without an artificial limb, that person will learn how to get around reasonably well by crawling, hopping, using a crutch, or finding some other means of non-bipedal locomotion. (Yes, now they could get a very sophisticated artificial leg.) I think it is inappropriate to say that they have evolved or developed a new means of locomotion, as all of those latent skills were present before the leg was lost. They just had not been required to be developed, but they could be refined and to some extent repurposed.***

We did not claim to have evolved an entirely novel mechanism of chemotaxis – the observed behavior does clearly rely of modification of the existing pathway. What we wanted to say is that the strategy of chemotaxis used by the evolved *cheR* cells is different from that used by the wildtype cells. While the latent ability to perform chemotaxis without receptor methylation might have been present in the wildtype cells, as suggested by the Reviewer (and it might have indeed existed before the modern chemotaxis pathway with receptor methylation system emerged), this strategy was not obvious and apparently not used. And even if this is indeed the case, such evolutionary reactivation of a latent behavioral strategy over a short period of time is very non-trivial. We thank the Reviewer for highlighting this point and we have now modified and expanded its discussion in the revised version of the manuscript.

***5) My conclusion from the points raised in the two preceding paragraphs is that the title and emphasis of the paper should be changed, and its claimed impact be put into perspective. It is an inelegant alternative title, and something better wording can no doubt but found, but more accurate would be “Loss of Adaptive Methylation by CheR Can Be Partially Compensated by Multiple Mutations Targeting Other Components of the Chemotaxis Signaling Pathway.”***

We changed the title to “Experimental evolution partially restores functionality of bacterial chemotaxis network with reduced number of components”. We have also clearly stated now in the Abstract that the evolved strategy is less efficient than the original one.

***6) It is also misleading to say that the system has lower complexity. The compensation for the loss of CheR, CheZ, or CheB, came through the accumulation of multiple mutations in other components of the chemotaxis and flagellar system that had to be finely balanced and likely made the system far less robust and flexible. The newly evolved chemotaxis system is almost certainly inferior to the one it replaced. Simply removing one component does not make the system less complex if multiple changes in the remaining components are required to have it function even somewhat effectively. Helicobacter pylori can get by with a simpler system because it lives in a rather simple environment.***

As mentioned before, we never claimed that the evolved chemotaxis system with a reduced number of components is superior to the ancestral one, and we absolutely agree with the Reviewer that the auxiliary components of the pathway are important for its robustness and versatility. We think that it could be argued that fewer components in a network indeed mean lower complexity, but to be more specific we have now changed the text to rather say “reduced number of components” instead of “reduced complexity”. We have further expanded the discussion of possible shortcomings of the newly evolved chemotaxis strategy.

**Minor editorial comments.
Line 565. The “circumvents” should be “circumvent.”**

Corrected, thank you.

***Lines 879-880. Rewrite “…where the corresponding individual chemotaxis gene was deleted, either before…or after evolution for 30 days.” The gene was not deleted before or after 30 days of evolution. The evolved strain with the deleted gene was evaluated before and after evolution for 30 days***.

Corrected, thank you.

***Reviewer #2: In this study, the authors investigated how E. coli adapts to the loss of key genes in the CheYZ pathway, which is critical for chemotaxis. They generated knockout strains, conducted experimental evolution, and analysed evolved mutants through sequencing, RNA-seq, and detailed characterization of swimming behaviour. The results showed that the evolved mutants partially restored certain aspects of chemotaxis, but not all, concluding that experimental evolution can lead to novel behaviour strategies.

I found the study to be very interesting, but I believe the authors may have overreached with their conclusions relative to the data presented and need to better align the strength of their interpretations with the evidence.***

We thank the Reviewer for finding our work very interesting and for suggestions on improvement of our manuscript.

***My comments:
Abstract - I am unclear about the authors' statement that E. coli is assumed to possess the 'minimal number of components for chemotaxis.' Does this simply mean that E. coli can perform chemotaxis and therefore has the necessary components, or is there an assumption that it has the minimal possible number of components? If the latter, this assumption should be justified, as it is not obvious why we should assume there is no redundancy in the system.***

This statement is explained in greater detail in the Introduction, but we agree that it might sound ambiguously. The assumption is, based on the previous mutational analysis and on the mathematical modeling of the pathway, that all activities are indeed required and there is no redundancy. We have now rephrased this sentence in the Abstract and additionally justified this assumption in the Introduction.

***Additionally, the authors claim that the complexity of the chemotaxis signalling pathway can be reduced through experimental evolution. However, it is not clear whether they have demonstrated a reduction in complexity or simply a reduction in the number of components. These are not synonymous, as complexity encompasses more than just the number of parts—it includes interactions, regulation, and system wide outputs. The authors should clarify their interpretation of 'complexity' and explain how their claims align with the evidence provided."***

We think that it could be argued that fewer components in a network could indeed mean lower complexity, particularly since we do not assume that the experimental evolution led to the emergence of novel, more complex, connectivity within the network. But to be more specific we have now changed the text, the title and the Abstract to rather say “reduced number of components” instead of “reduced complexity”.

***Line 152 can the authors please rewrite to make this a bit clearer, for example perhaps explain the phenotype of the mutants first. Then in separate sentence explain the phenotype of the evolved strains, including some comment on the variation between replicates and also some statistical reporting on the difference between the outcomes for different mutants.***

We revised the text accordingly.

***Line 156 what is a core component. Is it a component that, after it has been deleted, evolution cannot restore the function? or is there some other definition.***

We now clarify the definition of the core components in the text.

***Line 185 It is not clear to me what conclusions are being referred to here. I presume the result was consistent with what was observed in this study, but not exactly the same, so please explain what the result was that was observed, and why is this worth mentioning.***

The statement has been rephrased to improve clarity.

***Line 191 “correlated well” is a bit vague - can the authors please supply R values and P values. Also, it would be interesting to know if this is something that has been observed before. In other words is it typical that tumbling correlates with spreading.***

We now show such correlation as inset in Figure 2B, and also cite a very recent paper (Vo et al, 2025), published after the submission of our manuscript, that draws a similar conclusion about the optimality of wildtype tumbling bias.

***L198 the author's conclusion is too strong here. This is interesting, but circumstantial evidence - you do not consistently see elevated fliC expression, and you have not done the molecular genetic follow up experiments to really nail down the result. That is fine, but I think the authors should say that these data suggest or support the hypothesis rather than using words like “confirm” or “likely”.***

The text was rephrased accordingly, also referring to the previous publications that support this hypothesis. We further included a graph showing the correlation between swimming speed and gene expression (Figure 2D Inset), which is consistent with this conclusion.

***L223 I am not sure what this sentence means. “These mutations may promote the active state of Tsr or Tar, contributing to the suppression of low receptor activity that is characteristic for the cheR strain.” It makes sense for me until it gets to the part “that is characteristic for the cheR strain.” Can the authors please write to make the meaning more clear? – perhaps unpacking this in to more that one sentence will help.***

We rephrased this text to make it clearer.

***L223 to L230 – this section has quite a few ideas that are not quite fully explained. For example this sentence “Mutations in tsr may have been preferentially selected over those in tar either because the first gradient followed by chemotactic cells on TBSA plates is formed by the Tsr ligand serine (28), or because cells have higher levels of Tsr compared to Tar under our growth conditions.” is difficult to understand. Please break this up into multiple sentences and make sure there is enough information for the uninitiated to understand.***

We revised the text to better clarify our conclusions. We also deleted the second part of this sentence, following the suggestion of the Reviewer #3.

***L224 – The authors write that “These mutations may promote the active state of Tsr or Tar,”. This seems very unlikely to be true for the TAR gene. There is a very important difference between the TSR and the TAR mutations - basically all of the TAR mutations cause a major loss of function, either a frameshift or an early stop. This tells us that what has been selected in these strains is a loss of function of this protein. This is pretty convincing evidence since the TSR mutants are all amino acid substitutions, in contrast. I think the authors should discuss what this means and modify their hypothesis about the impact of mutations in these two genes. Or alternatively explain in the response what I'm missing.***

We apologize if this was unclear, but this line refers to mutations in the evolved *cheR* lines, whereas the loss-of-function mutations in tar are found in *cheZ* lines. We now modify the text to make it clearer.

***L251 – does this refer to all deltaCheZ lines? Or all lines in the study? Also did the authors start the study with clones? This is typical in evolution experiments, to reduce the chance that parallel evolution gets mistaken for standing genetic variation. Were there any other instances where the same exact mutation was seen in multiple different lines? Even if they were at a low frequency, the fact remains that they they spread to a high frequency during the course of the experiments suggesting that they were selected. What is the point that the authors are making here, in other words what are they suggesting when they point out that these particular mutants may have been already in the population before the study?***

We indeed assume that these mutations were already present at low frequency in the population prior to the evolution, as was already stated in the previous version of the manuscript. We now modified this text to explicitly refer to standing genetic variation. We now explain in the Methods that the starting cultures for all evolution experiments were started from the glycerol stock and not from different clones. For the purpose of our study, standing variation was not considered to be a problem and might have been even advantageous, potentially increasing the pool of mutations that could be selected. However, we also note in the Methods that the presence of SNPs was tested at different stages of selection using Sanger sequencing, and none of the SNPs was observed in the parental strains at a detectable level.

***Methods: I would like the authors to provide more information about the provenance of their mutants - were they made for the study or were they made some time ago? In other words, has there been time to accumulate mutations in the lab, since they were made. Also, did the authors sequence the constructed mutants, or just compare the evolved strains to the ancestor? And also what was the process of setting up the experiment was each replicate line a separate clone or were replicate lines founded from the same clone (or at least, single colony). These details will be important for people doing evolution experiments and also for understanding the results of the sequencing.***

We now elaborate of these points in the Methods. In brief, the knockout strains used in our study were constructed and described already in previous publications, as specified in S4 Table where the strains are listed. We did not specifically sequence the deletion strains. But since multiple evolved lines for each deletion strain were sequenced, the absence of common mutations that would appear in all lines for a particular strain confirms that they do not carry secondary mutations compared to the ancestral strain. Moreover, we have validated the absence of the identified SNPs in the parental strains by Sanger sequencing.

***There is a method described for making point mutations - is this method for making the knockouts?***

As mentioned above, the knockouts were constructed and described in previous studies.

***Figure 1 - the first panel depicting the different components of signal transduction in motility are too small and it's not clear which components correspond to which gene. For panel B the series of photos purporting to show different levels of chemo tactic capacity are a bit unclear. Panels C DE are organised in an odd way - shouldn't you just show the deletion mutant 1st and then show the results of the evolution experiment? I found the arbitrary use of color a little confusing - why some panels blue and some red, and then others in gray?***

We now enlarged the respective panels of this figure and made the presentation uniform. We added space between individual groups of strains, highlighting that, for each group, the deletion mutant is indeed showed first, followed by the evolved lines (we think that this is a better representation than showing all deletion mutants first, because we primarily compare each set of evolved lines to the parental deletion strain). We also explained the usage of colors in the figure legend (the colors in C-D match the colors in A).

***Reviewer #3: The authors deleted the chemotaxis genes from the E. coli genome one by one and then examine how chemotaxis might be recovered via evolution. They uncover several compensatory mechanisms that reestablish the ability to climb chemical gradients, revealing what aspects of the pathway are essential and which one are not. Notably they show that chemotaxis can take place without adaptation mechanisms by only controlling the effective diffusion coefficient of cells (mean tumble bias) as a function of the absolute concentration of ligand, decreasing it as ligand concentration goes up.

The powerful E. coli chemotaxis system is ideal for this study and experiments are carried out carefully. Some of the analysis needs some work, especially the measurements of tumble bias, which in wild type look very different from what is measured in other studies. Overall this is an excellent study that should be of interest to the broad readership of PLoS Genetics.***

We thank the Reviewer for the appreciation of our study and for helpful suggestion on improvement of our manuscript.

***Main comments:

Figure 1C and elsewhere: How is the spreading ring size normalized? It appears that the authors divide the size of the evolved mutants’ rings by that of the WT ring. However, is the WT strain also evolved, or is only the non-evolved WT used for normalization? The authors previously demonstrated that the spreading rate of RP437 increases significantly after ~6 rounds of evolution (Ni et al., 2017).***

The values are always normalized to the spreading of non-evolved wildtype RP437 (same as shown in Fig. 1B). We now specifically state this in the figure legend. In our opinion, it is much more straightforward to use the non-evolved RP437 as a reference strain, since different evolution lines of the wildtype cells in our previous study have improved spreading to different degrees, and it would be a rather arbitrary decision which of them to use. But we entirely agree that comparison with the previous evolution of RP437 is relevant. We did refer to that previous paper a number of times throughout the manuscript, but in the revised version of the manuscript we have now also explicitly mentioned the enhancement of spreading for the evolved RP437 when referring to the results in Figure 1C in the text.

***Figure 2A: Line 189: unlike for CheR and CheZ, for CheB it is not really the case that most evolved lines reduced tumbling to WT levels. Can the authors comment on this? Why is CheB different? Is it due to the extra regulation of CheB via phosphorylation?***

We agree that this is interesting but do not have a simple answer to this question. One possible explanation could be that the initial tumbling bias of the ∆*cheB* strain is not as high as for the ∆*cheZ* strain, so it is closer to the optimal bias and the selection pressure to reduce it might be lower. It is also possible that the regulation of CheB activity by phosphorylation might play a role, as suggested by the Reviewer. Notably, this observation further supports our conclusions that the adjustment of tumbling can only partly account for the recovery of spreading (see our response to Reviewer #1), which we now specifically mention in the Results.

***Figure 2D: The promoter activity of fliA, the upstream regulator of fliC, decreases with increasing growth rate (Honda et al., 2022). Could the observed difference in fliA promoter activity be explained by a slower growth rate in the evolved strains? Did the authors measure growth rate in these strains, and if so, does it correlate with spreading rate?***

We thank the Reviewer for this suggestion. The Reviewer is correct, but the dependence of *fliA* (or *fliC*) activity on the growth rate in minimal medium supplemented with different carbon sources, as reported by Honda et al (and also in earlier studies, including one by our group, Ni et al, 2020), is the consequence of carbon catabolite repression, and it must not hold for the growth in rich medium (TB).

Nevertheless, in our previous studies, Ni et al., 2017 (mentioned above) and Lisevich et al., 2025 (mentioned below), we indeed observed a clear anticorrelation between the growth rate and *fliC* activity in TB. In those cases, however, the causality was the opposite, with higher flagellar expression causing growth rate reduction, because of high fitness cost of flagella production.

We now show the data for the growth rate of the evolved strains (new Figure S3) and discuss it in the text. However, while the growth rate of all evolved strains was indeed lower, there was no simple correspondence between growth and *fliC* reporter activity for individual strains.

***Figures 2C and 2D: Strain R1 exhibits ~2.5× higher fliC expression than strain R2, yet both strains have nearly identical run speeds. The authors previously showed a strong correlation between fliC expression and swim speed (Lisevich et al.). Could this indicate that R2 has reached the fliC expression threshold above which the run speed plateaus? If so, do the authors believe the increased fliC expression in the evolved strains provides any functional advantage?***

We thank the Reviewer for this suggestion, too. The increased *fliC* expression above that in RP437 does increase swimming speed (as was observed already in Ni et al., 2017 and also confirmed in Lisevich et al., 2025), thus providing functional advantage. But the Reviewer is right, and this effect saturates at even higher expression levels (and the situation is different in *E. coli* MG1655 that was primarily studied in Lisevich et al., 2025, where the native expression is already close to saturation). Comparison of these *fliC* reporter activity values with those obtained in Lisevich et al. indeed confirms that R1 has higher expression level than would be necessary to achieve the maximal run speed. To illustrate it, we now plotted the run speed values as a function of *fliC* reporter activity for all strains (new Figure 2D Inset).

It is possible that the increase in *fliC* expression in R1 may be an evolutionary “overshoot”, which could be expected in experimental evolution that relies on a small number of discrete mutations. Such elevated expression above the saturation of swimming was also observed in several cases in our previous study (Ni et al., 2017) for the evolution of the wildtype. We now mention this in the Discussion.

In principle, it is also possible that *fliC* activity above the saturation of swimming velocity might provide additional benefit - e.g., leading to larger and potentially more sensitive chemosensory clusters. But we are reluctant to speculate about this in the text without further evidence.

***Lines 229-230: The authors state that E. coli has higher levels of Tsr than Tar under their growth conditions. However, their 2010 study showed that RP437 has more Tsr than Tar molecules only at ODs < 0.1. Given that cell density is presumably high on agar plates, do the authors have Western blot data to support this claim?***

We do not think that it is straightforward to match the growth phase in the spreading ring in soft agar to that in the batch culture. This statement to which the Reviewer refers was only meant as one of possible explanations for the preferred accumulation of mutations in Tsr (compared to Tar), but we do not really claim it and it is anyway not our preferred explanation. We have thus removed it in the revised version of the manuscript.

***Line 251 and Table S3: The evolved ΔcheZ strains all carry tar mutations, but none have tsr mutations. If tar translation is completely disrupted, cells would predominantly express Tsr receptors, leading to lower kinase activity. Could the enhanced spreading rate in these strains be attributed to increased serine sensitivity (due to Tsr dominating the kinase response) combined with reduced kinase activity (due to the absence of Tar), leading to lower CheY-P levels compensating for the lack of CheZ?***

These are indeed two possible effects of Tar disruption mentioned by the Reviewer, the reduction of the overall receptor number and thus of activity of the receptor-associated CheA and the increase in sensitivity of more homogeneous Tsr cluster to serine. We believe that both can contribute to the enhanced spreading of *cheZ* lines, and we now mention them in this section of the Results. We thank the Reviewer for bringing this up.

***Lines 251-256: Unlike for the DeltaCheB, the authors do not provide a rational for the mutation they observe in the deltaCheZ lines. Why deletion of CheZ that results in high CheY-P would lead to mutation that interrupt Tar translation? How does that lead to reduction of kinase activity.***

See above. We absolutely agree with the hypotheses of the Reviewer and mention them in the revised version of the text.

***Likewise is the mutation in the P1 domain of CheA compensating for the reduced dephosphorylation of CheY by reducing phosphorylation rate?***

Yes, this is also our hypothesis. We apologize for not phrasing it explicitly. It is now corrected.

***Line 307: the authors conclude that the CheB activity is reduced. Is there direct evidence of that?***

That the observed mutations in CheB likely reduced its catalytic activity was discussed in the earlier section of the Results (lines 231-243 in the previous version of the manuscript). At the point of the manuscript to which the Reviewer refers, we rather stressed that some level of CheB activity is nevertheless required for spreading of the R1 line, because the deletion of *cheB* gene in this line abolishes spreading. We have now measured the impact of this CheB mutation, as well as the mutation in Tsr, in the wildtype background on the pathway response, directly confirming that it reduces the rate of adaptation (new Figure S5).

***Figures 4 and S4: Many evolved mutants form a ring when chemotaxing toward serine (Figure S4), yet none form a ring toward MeAsp (Figure 4), whereas the WT forms a ring in both cases. Why is that?***

We believe that this might be related to steeper gradients that are present in soft agar in the case of serine, because serine is metabolized inside the spreading colony. As the WT is better chemotactic, it can form rings in both cases, whether the less chemotactic strains can do it only in a very steep gradient. Alternatively, it could also be due to different impact of the mutations in *tsr* in these lines on the responses to serine and MeAsp, as indicated by our new FRET measurements (new Figure S5; see previous point). Finally, better spreading in serine might also be related to the fact that our selection for spreading occurs in gradients of serine. We now discuss these in the text (Discussion).

***Figure 6 and S8: the distribution of tumble bias is extremely wide in Figs 6C, S8B and even for the wt RP437 (Fig s8C): the distribution is almost uniform up to 0.6. Several previous studies have shown that the distribution of tumble bias in that same strain is typically much narrower with a peak around 0.2-0.3 with almost no cells with tumble bias above 0.5. Is this an artefact of the tracking or analysis algorithm? Did the authors count also cells that barely move, or count multiple tumbles within one effective tumble? It is difficult to assess as not much information is provided about the quantification of tumble bias from individual tracks.***

We believe that this wide distribution of the tumble bias is indeed a real property of our wildtype RP437 strain. As a comparison, we now provide measurements for *E. coli* MG1655 using the same tracking and analysis algorithm, which indeed show markedly narrower distribution (new Figure S10C). We now mention this in the text. We have made the description of cell tracking in the Methods section more detailed.

***Modeling. does the modeling predict a maximal stimulus where the population can no longer chemotax? Presumably, once the kinase activity is maximally suppressed, there are no longer position-dependent changes in tumbling rate.***

Indeed, we expect the *cheR*-deficient R1 cells to have a narrower range of concentration where they can sense a gradient, because at high attractant concentration their response will become saturated. Having said that, we also now performed additional measurements of the tumbling bias for the R1 cells exposed to MeAsp for several hours, and observed a very slow but reproducible and significant adaptation of their tumbling bias (new Figure 6E). Although our model suggests that such slow adaptation will not directly improve the chemotactic drift in the gradient (it is too slow for that), it may play an important role in counteracting the saturation effects mentioned by the Reviewer. We now discuss this in the text.

***Figure S7E. the dynamic range of the kinase response appears larger than the wild-type, which is in line with the interpretation that chemotaxis is made possible by extending the concentration range over which tumble bias varies. Do the authors know if the receptor or kinase mutations affect the cooperativity of the chemoreceptor arrays? How do these mutations affect WT receptor arrays?***

Our model indeed suggests that the absence of saturation of the R1 response even at high concentrations of MeAsp is important for their chemotaxis. To better highlight this result, we now moved these data to the main text (new Figure 6B) and extended their discussion. We have also performed additional FRET measurements (mentioned above) for the *tsr* mutation introduced into WT cells, as suggested by the Reviewer (new Figure S5). These data suggest that this mutant shows a steeper response to serine (which might be helpful in following serine gradients in soft agar) while the response to MeAsp is largely unaltered although possibly slightly less steep. We discuss these data as well.

***Minor comments:
Line 120: drop the word nutrients? E.g. aspartate and serine are not really nutrients***

*E. coli* can metabolize both aspartate and serine (e.g., ref #32 in the revised manuscript), so we think that calling them nutrients is justified. The consumption of serine is now explicitly mentioned.

***Figure 1: Scale bar in panel B is missing. C-E: What does 2, 3, and 4 mean in e.g. B2 B3 B4. Are these are replicates of te evolutionary experiment starting with the same ancestor?***

The scale bar has been added; we apologize for missing this in the previous version of the manuscript. The meaning of numeration is now better explained in the figure legend (these are indeed independent replicates of evolution starting with the same ancestral deletion strain) and the description of the evolution experiment in the Methods is improved.

***Line 191: claim that tumbling correlates with spreading in TBSA, but no correlation is calculated in the text or the figure caption***

As suggested by the Reviewer, we now added a plot showing the anticorrelation between the deviation of the tumbling bias from the wildtype value and spreading in TBSA and calculated the Spearman’s rank correlation coefficient between these variables (─ 0.51) (Figure 2B Inset).

***Line 193: Claim ‘nearly all’ evolved strains have increased swim speed. Should clarify that this is w.r.t. the deletant strains, because the significance in Figure 2C is calculated w.r.t. the wild-type.***

For consistency with other plots, we have now recalculated the significance values w.r.t. the parental deletion strains in this plot.

***Figure 3C: the text makes reference to receiver domains, and methylesterase domains. Could these be labeled like the receptor domains in 3AB?***

We have now added the suggested labels and we thank the Reviewer for this suggestion.

***Figure 4B: I’m a bit confused on how this bias is calculated. The authors say it’s the ratio between ‘spreading’ up and down the gradient, but how is this spreading calculated? Is this just the furthest point reached in either direction, or does this take into account the overall shape of the cell density?***

We have now added the definition of the spreading bias in the figure legend; we also changed the axis label from “bias” to “spreading bias” to improve clarity.
