# Supplementary material for: Experimental evolution partially restores functionality of bacterial chemotaxis network with reduced number of components
Source: PLoS Genet. 2025 Jul 10;21(7):e1011784. doi: 10.1371/journal.pgen.1011784 (PMC12270135; doi:10.1371/journal.pgen.1011784)
Supplement: Attachment — Submitted filename: Kargeti Response to Reviewers.docx [file pgen.1011784.s021.docx]

Response to Reviewers

Reviewer #2: The authors have addressed all of my concerns, and their rewrites make the paper much clearer. I have no future comments.

Reviewer #3: The authors have done a great job at addressing my questions in detail and have updated the figures accordingly.

*We thank both Reviewers for their carefully assessment of the manuscript and insightful suggestions for its improvement. We are very glad to see that we have been able to satisfactory address the Reviewers’ questions. We have now made last minor revisions to address the remaining questions of the Reviewer #3.*

I have a few remaining minor questions:

The authors now compare the tumble bias of MG1655 and RP437, saying that the experimentally measured tumbling bias of the wildtype RP437 strain was “markedly broader than that of the canonical E. coli MG1655 strain (Figure S10C)”. However, in Figure S10c the distributions for RP437 and MG1655 look quite similar. The use of a log scale for the vertical axis might be misleading when talking about the width of these distributions. Could the authors also plot them with the vertical axis on a linear scale? In general, in might be more informative to plot the tumble bias distributions using a linear scale for the vertical axis throughout the paper.

*To better highlight the difference between MG1655 and RP437 strains, we now additionally plotted the distributions of their tumbling biases on linear scale, as suggested by the Reviewer (Supplementary Figure S10C). Since the cells with intermediate levels of tumbling are important for the chemotactic response at all MeAsp concentration, we kept the log scale distributions for the R1 cells in the main Figure 7, but we now show the same disctribution on a linear scale in Supplementary Figure S10B. Note that the datasets underlying Supplementary Figure S10C now contain a larger number of trajectories, which we added from additional replicates to further probe the differences in distribution widths. Our RP437 strain indeed shows a wider distribution than MG1655, although it is outside of the scope of the study to investigate how significant this difference is, and we therefore removed “markedly” from the sentence.*

There seems to be a high density near zero tumble bias in the RP437 distribution, which is surprising for the wild type RP437. Could this be an artifact? A minimal track duration of 1 s is very short. A typical value needed for accurate determination of tumble bias is 10 s. Also are some of the cells circling near the surface, which suppresses tumbles? Finally, if tracks have different lengths, the tumble bias of each track should be weighted by the duration of the track when generating the distribution to avoid over-counting low tumble bias cells.

*The Reviewer is absolutely right, and the high density of zero tumbling bias indeed comes in part from tracks of short durations. It primarily results from tracks in the bulk of the channel, in which a motile cell zooms through part of the field of view without tumbling. With the larger dataset, the peak became slightly less prominent, but did not disappear. Because we do not want to hide these cells, we kept the trajectory duration threshold as before but now clarify in the legend of Supplementary Figure S10C that this zero-tumbling peak corresponds to short tracks.*

*Additionally, trajectories were indeed weighted by their duration when generating the distributions, as suggested by the Reviewer, already in the previous version of the anaylysis. We apologize for not specifying explicitly in the previous version of the manuscript and we stated in now in the Methods section.*
